# Impaired HDL cholesterol efflux in metabolic syndrome is unrelated to glucose tolerance status: the CODAM study

**DOI:** 10.1038/srep27367

**Published:** 2016-06-08

**Authors:** Wijtske Annema, Arne Dikkers, Jan Freark de Boer, Marleen M. J. van Greevenbroek, Carla J. H. van der Kallen, Casper G. Schalkwijk, Coen D. A. Stehouwer, Robin P. F. Dullaart, Uwe J. F. Tietge

**Affiliations:** 1 Department of Pediatrics, University of Groningen, University Medical Center Groningen, Groningen, The Netherlands; 2 Top Institute Food and Nutrition, Wageningen, The Netherlands; 3 Laboratory for Metabolism and Vascular Medicine, Department of Internal Medicine, Maastricht University Medical Centre (MUMC), Maastricht, The Netherlands; 4 CARIM School for Cardiovascular Diseases, Maastricht University, Maastricht, The Netherlands; 5 Department of Endocrinology, University of Groningen, University Medical Center Groningen, Groningen, The Netherlands

## Abstract

Type 2 diabetes mellitus (T2DM) and metabolic syndrome (MetS) increase atherosclerotic cardiovascular disease risk. Cholesterol efflux capacity (CEC) is a key metric of the anti-atherosclerotic functionality of high-density lipoproteins (HDL). The present study aimed to delineate if T2DM and MetS cross-sectionally associate with altered CEC in a large high cardiometabolic risk population. CEC was determined from THP-1 macrophage foam cells towards apolipoprotein B-depleted plasma from 552 subjects of the CODAM cohort (288 controls, 126 impaired glucose metabolism [IGM], 138 T2DM). MetS was present in 297 participants. CEC was not different between different glucose tolerance categories but was lower in MetS (*P* < 0.001), at least partly attributable to lower HDL cholesterol (HDL-C) and apoA-I levels (*P* < 0.001 for each). Low grade inflammation was increased in IGM, T2DM and MetS as determined by a score comprising 8 different biomarkers (*P* < 0.05-< 0.001; n = 547). CEC inversely associated with low-grade inflammation taking account of HDL-C or apoA-I in MetS (*P* < 0.02), but not in subjects without MetS (interaction: *P* = 0.015). This study demonstrates that IGM and T2DM do not impact the HDL CEC function, while efflux is lower in MetS, partly dependent on plasma HDL-C levels. Enhanced low-grade inflammation in MetS may conceivably impair CEC even independent of HDL-C and apoA-I.

It is well recognized that both type 2 diabetes mellitus (T2DM) and the metabolic syndrome (MetS) confer a substantial increase in the risk of atherosclerotic cardiovascular disease (CVD)^1^,^2^,^3^,^4^. Increased CVD risk in these conditions is at least in part related to low plasma levels of HDL cholesterol (HDL-C)^4^,^5^, in line with the adverse impact of low HDL-C in general population studies^6^,^7^. More recently, a shift in concept has been proposed suggesting that impaired HDL function rather than low HDL-C per se may explain HDL-associated risk in these conditions^8^,^9^. Indeed, among other important HDL functions, its ability to stimulate cellular cholesterol efflux may predict presence^10^ and future risk^11^,^12^ of CVD, even independent of HDL-C levels per se.

In the context of HDL function cholesterol efflux represents a key metric^9^,^13^. Cholesterol efflux comprises most relevantly the mobilization of cholesterol from macrophage foam cells and is the first step in the atheroprotective reverse cholesterol transport (RCT) pathway^13^,^14^. Thus far, variable changes in the cholesterol efflux capacity of HDL from T2DM patients have been reported. While some found decreased efflux^15^,^16^,^17^,^18^,^19^, others reported unchanged^20^ or even increased^21^ HDL efflux capacity in T2DM. However, such studies have mostly been carried out in relatively small and selected groups of T2DM patients, and it is still unclear whether altered HDL glycation due to hyperglycemia adversely affects its ability to promote cholesterol efflux^16^,^21^,^22^,^23^. Of further importance, enhanced low-grade systemic inflammation is a well known feature of T2DM and MetS^24^. In turn, inflammation is thought to be a pathophysiologically important determinant of an impaired efflux functionality of HDL and subsequently also reduced RCT^25^,^26^.

The aim of the present study was (i) to assess in a large cohort of well-characterized subjects whether the degree of glucose tolerance impacts on the cholesterol efflux capacity and (ii) to evaluate potential factors determining such changes.

## Results

Five hundred fifty two subjects from the CODAM cohort participated in the present study, of whom 288 had normal glucose metabolism (NGM), 126 impaired glucose metabolism (IGM) and 138 T2DM. The respective clinical and laboratory characteristics according to glucose tolerance status are shown in Table 1. T2DM subjects were slightly older compared to NGM subjects, had a higher body mass index (BMI) and were classified with MetS more frequently. A positive history of CVD was most prevalent in T2DM subjects. T2DM subjects also had a higher blood pressure, and used antihypertensive medication as well as glucose lowering drugs more frequently. Plasma triglycerides were highest, whereas HDL cholesterol and apolipoprotein (apo) A-I levels were lowest in T2DM. The low-grade inflammation score was highest in T2DM and lowest in NGM subjects. Of note, cholesterol efflux capacity did not differ significantly among the glucose tolerance categories (Table 1). There was also no difference in cholesterol efflux capacity between glucose tolerance categories after adjustment for age and sex (*P* > 0.19; data not shown). Additionally, cholesterol efflux capacity was not different in NGM subjects (n = 288; 1.35 ± 0.30) vs. subjects with IGM or T2DM combined (n = 264; 1.32 ± 0.29, *P* = 0.31).

When the participants were categorized according to the presence and absence of MetS, individuals with the MetS expectedly had higher blood pressure, BMI and waist circumference, more frequently used antihypertensive drugs, and had a poorer glycemic status (Table 2). Prevalent CVD and use of lipid-modifying medication was more frequent in participants with MetS. As expected, MetS subjects had higher triglycerides and apoB levels as well as lower HDL-C and apoA-I levels. The low-grade inflammation score was also higher in MetS. Notably, the cholesterol efflux capacity was significantly reduced in MetS subjects compared to those without MetS (Table 2, *P* < 0.001).

In the whole study population, cholesterol efflux capacity was lower in men than in women (1.30 ± 0.28 vs. 1.38 ± 0.31, respectively, *P* = 0.002), and in current smokers than in non-smokers (1.27 ± 0.28 vs. 1.35 ± 0.30, respectively, *P* = 0.014); these differences disappeared after adjustment for HDL cholesterol (*P* = 0.93 and *P* = 0.18, respectively). Cholesterol efflux capacity was not significantly different between subjects with and without a history of CVD (*P* = 0.27), subjects using and not using glucose-lowering drugs (*P* = 0.17), lipid-modifying drugs (*P* = 0.07) or antihypertensive medication (*P* = 0.22). Also when dividing the participants according to MetS and prevalent CVD, cholesterol efflux capacity did not differ between the respective groups according to CVD status (Supplementary Table 1).

Given sex-dependent differences in cholesterol efflux capacity, we carried out sex-adjusted regression analyses to determine the relationships of cholesterol efflux capacity with clinical and biochemical variables. There was a strong positive correlation of cholesterol efflux capacity with HDL cholesterol and apoA-I in the whole study population (Table 3). Similar correlations were observed in the separate glucose tolerance groups, and in subjects with and without MetS (interactions: *P* > 0.33 for all). Cholesterol efflux capacity was positively related to alcohol intake in the whole group and in IGM, T2DM and MetS subjects separately. Cholesterol efflux was unrelated to systolic blood pressure, waist circumference, non-HDL cholesterol, triglycerides, plasma glucose and HbA1c (Table 3). In all subjects combined, as well as in individuals with either T2DM or MetS, cholesterol efflux capacity negatively correlated with the low-grade inflammation score (Table 3). Such a relationship was neither observed in NGM and IGM subjects, nor in subjects without MetS.

We subsequently tested whether cholesterol efflux capacity was determined by the presence of MetS taking account of glucose tolerance status. In age- and sex-adjusted multivariable linear regression analysis, cholesterol efflux capacity was negatively related to MetS independent of glucose tolerance status (Table 4, model 1). This relationship remained statistically significant after additional adjustment for clinical covariates (Table 4, model 2). This analysis confirmed that there was no significant association of cholesterol efflux capacity with glucose tolerance status.

We next determined the extent to which cholesterol efflux capacity was independently associated with HDL cholesterol, apoA-I and low-grade inflammation in all subjects combined, as well as in NGM, IGM and T2DM subjects, and in subjects with and without MetS separately. Cholesterol efflux capacity was positively related to HDL cholesterol and - in parallel analyses - to apoA-I in the whole study population and similarly in the three glucose tolerance groups (Supplementary Table 2). These robust relationships were not substantially altered after further adjustment for clinical covariates. In these multivariable regression analyses, a relationship of cholesterol efflux capacity with the low-grade inflammation score was virtually absent in NGM subjects. In T2DM subjects, cholesterol efflux capacity tended to be inversely associated with the low-grade inflammation score (Supplementary Table 2). When similar analyses were performed in subjects with and without MetS separately, cholesterol efflux capacity was observed to be independently and inversely associated with the low-grade inflammation score in subjects with MetS, but not in subjects without MetS (Table 5; interaction: *P* = 0.015). Finally, we assessed the possible contribution of the individual inflammation markers to cholesterol efflux capacity in MetS subjects. In age-, sex- and HDL cholesterol adjusted analyses, cholesterol efflux capacity was independently and inversely associated with either haptoglobin, ceruloplasmin, serum amyloid A (SAA) and high-sensitivity C-reactive protein (hs-CRP) (*P* < 0.03 for each), but not with the other inflammation markers (*P* > 0.15 for each) (data not shown). Similar associations were found upon controlling for apoA-I instead of HDL cholesterol (data not shown). Combined these data demonstrate that low-grade inflammation in MetS patients may confer decreased cholesterol efflux independent of plasma HDL-C levels.

## Discussion

The present cross-sectional study investigated the impact of glucose tolerance status and the presence of MetS on a key functional metric of HDL, namely cholesterol efflux capacity, in a large cohort of clinically well-characterized Caucasian subjects selected for high cardiometabolic risk. Our results demonstrate that impaired glucose metabolism or established T2DM do not translate into a decreased cholesterol efflux capacity. On the other hand, the presence of MetS was associated with diminished cholesterol efflux capacity, which largely coincided with decreased HDL cholesterol and apoA-I plasma levels. Interestingly, our data also point towards a role of enhanced low-grade systemic inflammation in decreasing cholesterol efflux in MetS subjects, even independent of plasma HDL-C and apoA-I levels.

The impact of impaired glucose tolerance and T2DM on cholesterol efflux towards plasma/serum or isolated HDL is not fully clear. Thus far, mostly in smaller sized studies, T2DM was variably associated with changes in the cholesterol efflux capacity of HDL. Thereby, on the one hand reduced cholesterol efflux capacity was reported using a wide range of cell systems and extracellular acceptors, specifically LpA-I particles in an adipocyte cell line^15^, whole plasma or serum in Fu5AH hepatoma cells^17^,^19^, HDL3 in mouse peritoneal macrophages^16^, or serum in transfected cell systems to specifically evaluate SR-BI- and ABCG1-mediated efflux^18^. However, in a system using Fu5AH cells efflux towards plasma from T2DM patients was unchanged compared with control subjects with similar HDL cholesterol levels^20^. On the other hand, with THP-1 macrophages, as also used in our present study, increased efflux rates towards whole plasma, apoB-depleted plasma and HDL isolated by ultracentrifugation were seen in T2DM patients than in controls^21^. A separate approach reported increased cholesterol efflux in T2DM using whole plasma and human skin fibroblasts, but only in hypertriglyceridemic subjects^27^, whereas cholesterol efflux was unchanged in MetS subjects^28^. Another study confirmed greater efflux rates in hypertriglyceridemic compared with normotriglyceridemic patients using apoB-depleted plasma in BHK cells^29^. However, in our study comprising a large number of subjects with various degrees of glucose tolerance and wide variation in plasma triglyceride levels no association of efflux with plasma triglyceride levels were discernable. The reason for these experimental discrepancies is incompletely understood. It might conceivably be attributed to the different cholesterol acceptor systems used as well as the different cell lines used as cholesterol donors. In the interpretation of our study it should also be noted that metabolic control was in general adequate in the studied T2DM subjects, making that no conclusions can be drawn for efflux in the context of more severe hyperglycemia. It is important to point out that no gold standard is currently agreed upon for cholesterol efflux assays and that there is even no consensus on a preferred method to isolate HDL^9^. In our view, for relevance to CVD it is preferable to use for such studies a macrophage cell line, such as THP-1 or J774^10^, and apoB-depleted plasma^10^ or isolated HDL to optimally discern HDL efflux functionality. One advantage of apoB-depleted plasma is that it still contains preβ-HDL, the major acceptor for ABCA1-mediated efflux^9^. On the other hand, HDL isolated by e.g. ultracentrifugation does not contain plasma components such as albumin that could potentially affect efflux. Furthermore, for the clinical consequences of diabetes also the effect of the disease on macrophages should be taken into account, since very high glucose concentrations can adversely affect the activity of known efflux transporters^30^,^31^.

Two smaller scale studies are also available on efflux in MetS patients^32^,^33^. One reported greater efflux capacity in 25 overweight MetS patients with insulin resistance compared to 22 overweight MetS subjects without insulin resistance^33^. In this study, however, no healthy controls were included and, of note, efflux to whole plasma was measured. In addition, although THP-1 cells were employed, these were, in contrast to our study, not loaded with modified LDL to induce foam cells. Further the THP-1 cells were treated with an LXR agonist which shifts efflux strongly to ABCA1, the transporter mostly responsible for efflux towards preβ-HDL, which was significantly higher in the insulin resistant subjects. The other study compared 35 MetS patients and 15 healthy controls using apoB-depleted serum and a non-macrophage cell line (BHK) transfected to express ABCA1 and ABCG1^32^. MetS patients had significantly higher ABCA1-mediated efflux related to the higher preβ-HDL levels in these patients. Serum has the additional disadvantage that to form incubations at room temperature for 20–30 min are required, a time during which subtantial HDL remodeling can occur^9^. Thus, the smaller study size and methodological differences likely account for the different results in comparison to our work.

A potentially important finding of our study is the inverse association between cholesterol efflux capacity and low-grade inflammation, which was particularly prominent in the context of T2DM and MetS. Inflammation is a condition associated with an impaired efflux function in animal models and humans^14^,^25^, and resolution of inflammation results in improved efflux^25^,^34^. Our current data imply that the efficiency of HDL cholesterol efflux capacity might be more strongly related to low-grade inflammation than to hyperglycemia per se. Over time, we have identified several metabolic parameters in the CODAM study that are related to low grade inflammation and insulin resistance. Some of those can be perceived to be able to affect HDL functionality and, as such, contribute to the increased cardiometabolic burden in the MetS. Such metabolic parameters include, for instance, circulating complement components^35^, which may be carried on HDL particles. However, we measured complement components in plasma, not on HDL. Likewise, we showed that iron metabolism is cross-sectionally and longitudinally associated with insulin resistance^36^ and might theoretically affect HDL functionality, although the biological relevance remains to be established^37^.

Of note, out of the 8 biomarkers combined in the inflammation score in our study specifically the hepatocyte-derived acute phase proteins haptoglobin, ceruloplasmin, SAA and hs-CRP were negatively correlated to HDL efflux capacity suggesting a prominent role of the liver in the modulation of HDL quality. Out of these four acute phase proteins most research has been carried out with respect to SAA. In inflammatory states, plasma SAA is almost exclusively bound to the HDL fraction^38^,^39^. Previously, it was shown that enrichment of HDL with SAA decreases its cholesterol efflux properties^18^,^40^, which also translates into lower *in vivo* reverse cholesterol transport in a rodent model^25^. However, it should be noted that in a small-scale study in humans no impact of SAA on THP-1-mediated efflux towards HDL from T2DM patients has also been reported^41^. On the other hand, SAA has been linked to impairment of other key functions of HDL such as the protection against oxitative stress^42^ and inflammation^43^. Haptoglobin was shown to bind to apoA-I^44^ and has been suggested to be a modulator of RCT as well^45^. For ceruloplasmin it was shown that it is associated with the HDL fraction in inflammatory conditions, playing a role in inhibiting LDL oxidation^46^,^47^. This contrasts with the other hepatocyte-derived inflammation markers like hs-CRP for which no direct mechanistic role in modulating HDL function is appreciated. A potentially important inflammatory biomarker, however, was not determined in the present study, namely myeloperoxidase. Myeloperoxidase-induced oxidative modifications of apoA-I have been shown to decrease cholesterol efflux and RCT^48^. Such a mechanism could conceivably contribute to the reduced efflux capacity in MetS patients.

Some potential limitations to this study should be considered. The chosen washout period for lipid lowering therapy was 14 days, which might not be sufficient to fully eliminate the effects of statins on lipoprotein metabolism. However, since patients with preexisting CVD were included, 14 days were considered the maximum period for safety reasons. In addition, a skewed lipoprotein profile, especially with high plasma triglycerides and low HDL-C levels was shown to affect influx of cholesterol into macrophages during efflux assays^49^, which is not accounted for by the methods employed in the present work or in previous studies by others dealing with cholesterol efflux capacity and cardiovascular disease^10^,^11^. Further, due to the design of our study there is an unavoidable overlap between MetS and glucose tolerance categories. We took this into account by multivariable analysis with mutual statistical adjustments for the presence of either category, but it cannot be formally excluded that a certain effect remains.

In summary, our present study demonstrates in a large cohort of well characterized subjects that insulin resistance and T2DM do not impact on cholesterol efflux, a key metric of HDL function. Rather, the presence of MetS translates into an impaired cholesterol efflux in close association with low HDL-C and apoA-I. Furthermore, we identified enhanced low-grade systemic inflammation as a relevant factor that may contribute to diminished HDL function independent of HDL-C and apoA-I levels specifically in the context of T2DM and MetS.

### Patients and Methods

#### Study population and design

The study population consisted of participants of the Cohort on Diabetes and Atherosclerosis Maastricht (CODAM). This study includes 574 subjects selected from a large cohort in the general population (*n* > 20,000) on the basis of an elevated risk of type 2 diabetes mellitus and cardiovascular diseases as described in detail elsewhere^35^,^50^,^51^. Inclusion criteria comprised Caucasian origin and age above 40 years, and, in addition, at least one of the following criteria: a positive family history of T2DM (first-degree relatives), BMI >25 kg/m^2^, a history of gestational diabetes, use of anti-hypertensive medication, postprandial glucose ≥ 6.0 mmol/L, or glucosuria. All study participants were extensively characterized with regard to their lifestyle as well as their cardiovascular and metabolic profile during two visits to the University’s metabolic research unit. The study protocol was approved by the Medical Ethics Committee of the University of Maastricht and the Maastricht University Medical Center, and all subjects gave written informed consent. The described methods were carried out in accordance with the approved guidelines.

Height was measured with a stadiometer and weight with electronic weight scales with subjects wearing light indoor clothes without shoes. BMI was calculated as weight in kilograms divided by height in meters squared (kg/m^2^). Blood pressure was measured twice in supine position and on the right arm after a 5 min rest with an oscillometric precision blood pressure instrument (Maxi stable 3, Speidel & Keller). Waist circumference was measured in standing position at the level midway between the lateral lower rib margin and the spina iliaca anterior superior.

The study subjects were asked to stop their lipid-modifying medication 14 days before and all other medication on the day before the visit to the University’s research unit (>80% adherence). The participants underwent an oral glucose tolerance test (except those with established T2DM), and were then categorized as having NGM, IGM and T2DM. IGM was considered in case of either impaired glucose tolerance and/or impaired fasting glucose levels^50^. Presence of the MetS was determined according to the National Cholesterol Education Program (NCEP) –Adult Treatment Panel (ATP) III definition as modified by the American Heart Association (AHA)/National Heart, Lung and Blood Institute (NHLBI)^52^. The exact distribution of the subjects over the different categories is given in Supplementary Table 3.

CVD was defined as self-reported myocardial infarction, coronary bypass surgery, percutaneous interventions, stroke or transient ischemic attack (through questionnaires) and/or the presence of signs of myocardial infarction (Minnesota codes 1–1 or 1–2) or ischemia (Minnesota codes 1–3, 4–1, 4–2, 4–3, 5–1, 5–2, 5–3 or 7–1) on a 12-lead electrocardiogram or an ankle-brachial pressure index <0.9 . For most of the cases (>75% of the subjects with self-reported CVD), the self-reports could be confirmed using available hospital registries.

Smoking habits as well as the use of medication were also assessed by questionnaires. Alcohol consumption was estimated by a validated food frequency questionnaire^53^.

#### Laboratory measurements

Blood samples were obtained by venipuncture after an overnight fast. EDTA blood was collected in pre-cooled tubes on ice, centrifuged at 3000 rpm for 15 min at 4 °C. EDTA plasma and serum aliquots were stored at −80 °C until use. Glucose was directly measured using routine procedures. Glycated hemoglobin (HbA_1c_) was determined by ion-exchange high-performance liquid chromatography (HPLC; Bio-Rad, Veenendaal, the Netherlands). Total cholesterol, HDL-cholesterol (HDL-C) and triglycerides were determined in EDTA plasma using the HDL-C plus assay, CHOD-PAP assay or the triglyceride GPO-PAP assay, respectively (Roche Diagnostics, Mannheim, Germany). LDL cholesterol was calculated with the Friedewald formula^50^. Plasma apoB and apoA-I were determined by immunonephelometric assays, using polyclonal rabbit anti-human apoB or apoA-I antiserum, and standards with the assigned values according to the International Federation of Clinical Chemistry (Behringwerke AG, Marburg, Germany)^50^.

Eight markers of low-grade inflammation were measured: hs-CRP, tumor necrosis factor-α (TNF-α), interleukin-6 (IL-6), IL-8, SAA, soluble intercellular adhesion molecule 1 (sICAM-1), ceruloplasmin and haptoglobin. The rationale for the specific selection of these markers and the respective assay systems used have been provided previously^51^. hs-CRP, IL-6, SAA and sICAM were measured by single biomarker techniques and with a multi-array (MA) detection system [Mesoscale Discovery, Rockville, MD USA]. The measures of the single biomarker techniques were realigned to the MA measures. IL-8 and TNF-α were determined in EDTA plasma using the MA detection system. Haptoglobin was measured in serum using the Tina-quant haptoglobin assay (Roche Diagnostics). Ceruloplasmin was measured with an immunoturbidimetric assay (Roche Diagnostics). A complete data set was available from 547 participants.

#### Determination of cholesterol efflux

HDL was isolated from EDTA plasma by precipitation of apoB-containing lipoproteins using polyethylene glycol (PEG 6000, Sigma, St Louis, MO) in 10 mM HEPES (pH = 8.0) as described previously^10^,^42^,^54^,^55^,^56^,^57^. Following 30 minutes centrifugation at 2200 *g* and 4 °C, the HDL-containing supernatant was transferred to a fresh tube and used the same day for cholesterol efflux measurements.

Cholesterol efflux capacity towards apoB-depleted plasma was studied using THP-1 human monocytes (ATTC via LGC Promochem, Teddington, UK) that were differentiated into macrophages by the addition of 100 nM phorbol myristate acetate^25^,^58^. Differentiated THP-1 macrophages were then loaded with 50 μg/ml acetylated LDL and 1 μCi/ml ^3^H-cholesterol (Perkin Elmer, Boston, MA) for 24 hours followed by equilibration for 24 hours in RPMI 1640 medium containing 2% bovine serum albumin^25^. After equilibration, efflux towards 2% apoB-depleted plasma was performed for 5 hours. Following table-top centrifugation to pellet cell debris, an aliquot of medium was counted to quantitate the effluxed cholesterol label. Meanwhile the cells were incubated for at least 30 minutes with 0.1 M NaOH at room temperature, whereupon the radioactivity remaining within the cells was determined by liquid scintillation counting (Packard 1600CA Tri-Carb, Packard, Meriden, CT). Efflux per well is expressed as the percentage of counts released into the medium related to the total dose of radioactivity initially present (counts recovered within the medium added to the counts recovered from the cells). Values obtained from control cells without added apoB-depleted patient plasma were subtracted to correct for unspecific efflux.

Cholesterol efflux capacity measurements were carried out in all respective patient samples at the same time to limit potential variation due to different assay conditions. All measurements were performed in duplicate. To correct for potential plate-to-plate variation, the same apoB-depleted control plasma was included on each plate at four different concentrations and individual values were normalized to values obtained with a pool of 2% apoB-depleted control plasma using these respective standard curves. Thus values given for efflux do not have a specific unit. The intra-assay CV of this method is 5.4%, the interassay CV is 7.9%.

#### Statistical analysis

SPSS 22 was used for data analysis. Results are expressed as mean ± SD or as median (interquartile range). Because of skewed distribution logarithmically transformed values of triglycerides and hs-CRP, TNF-α, IL-6, IL-8, SAA, sICAM-1 were used. A low-grade inflammation score was computed by averaging the *Z*-scores [(individual observed values − population mean)/population SD] of the 8 inflammation markers measured (hs-CRP, TNF-α, IL-6, IL-8, SAA, sICAM-1, ceruloplasmin and haptoglobin). The average value of the combined low grade inflammation score was used in the analyses. This composite score can be interpreted as eight repeated measurements of the same construct, i.e. inflammation, and thereby may reduce measurement errors. Moreover, the use of this inflammation score prevents multiple testing problems and is expected to reduce the influence of biological variability of each marker if assessed separately.

Differences in continuous variables among subjects with NGM, IGM and T2DM were determined by one-way analysis of variance (ANOVA) with subsequent Bonferroni method to correct for multiple comparisons. Differences between subjects with and without MetS were determined by Student T-tests for unpaired observations. Between-group differences in proportions were determined by Chi-square tests. Partial correlation coefficients were calculated taking account of sex. Multivariable linear regression analyses were carried out to determine the independent contribution of variables to cholesterol efflux. Interaction terms were calculated as the product terms of glucose tolerance status or the presence of MetS with HDL cholesterol, apoA-I and the low grade inflammation score. For continuous variables distributions centered to the mean were made by subtracting the individual value of the variable of interest from their group mean values to account for outliers. Interaction terms were considered statistically significant at *P*-values < 0.10, as proposed by Selvin^59^ and recommended by the Food and Drug Administration authorities. Otherwise, two-sided *P*-values < 0.05 were considered significant.

## Additional Information

**How to cite this article**: Annema, W. *et al.* Impaired HDL cholesterol efflux in metabolic syndrome is unrelated to glucose tolerance status: the CODAM study. *Sci. Rep.* **6**, 27367; doi: 10.1038/srep27367 (2016).

## Supplementary Material

Supplementary Information

## Figures and Tables

**Table 1:** Clinical characteristics, plasma glucose, glycated hemoglobin (HbA1c), plasma lipids, apolipoproteins (apos), low-grade inflammation score and cholesterol efflux in 552 subjects according to glucose metabolism status (normal glucose metabolism [NGM], impaired glucose metabolism [IGM] and Type 2 diabetes mellitus [T2DM]).

	NGM (n = 288)	IGM (n = 126)	T2DM (n = 138)	*P*-value
Age (years)	59 ± 7	60 ± 7	61 ± 6^b^	0.005
Sex (men/women)	173/115	75/51	91/47	0.45
MetS (yes/no)	92/196	87/39	118/20	<0.001
Systolic blood pressure (mm Hg)	135 ± 18	143 ± 18^c^	148 ± 19^c^	<0.001
Diastolic blood pressure (mm Hg)	80 ± 8	84 ± 9^c^	85 ± 10^c^	<0.001
Waist circumference (cm)	96 ± 11	101 ± 12^c^	105 ± 12c,e	<0.001
BMI (kg/m^2^)	27.5 ± 3.9	28.9 ± 4.3^b^	30.3 ± 4.7c,d	<0.001
Current smoking (yes/no/unknown)	60/222/6	25/99/2	26/108/4	0.94
Alcohol consumption (gram/day)	9 (2–23)	9 (1–23)	8 (1–21)	0.61
Cardiovascular disease (yes/no)	69/219	35/91	53/85a,d	0.008
Glucose lowering therapy (yes/no)*	0/288	3/123	68/70c,f	<0.001
Lipid-modifying therapy (yes/no)	46/242	25/101	35/103	0.069
Antihypertensive therapy (yes/no)	84/204	52/74^b^	80/58^c^	<0.001
Glucose (mmol/l)	5.3 ± 0.4	5.9 ± 0.5^c^	7.9 ± 1.8c,f	<0.001
HbA1c (%)	5.6 ± 0.4	5.8 ± 0.4^a^	6.8 ± 1.1c,f	<0.001
Total cholesterol (mmol/l)	5.22 ± 0.90	5.30 ± 0.93	5.18 ± 1.20	0.58
Non-HDL cholesterol (mmol/l)	3.94 ± 0.94	4.13 ± 0.95	4.12 ± 1.21	0.11
LDL cholesterol (mmol/l)	3.35 ± 0.87	3.37 ± 0.85	3.16 ± 0.87	0.084
HDL cholesterol (mmol/l)	1.27 ± 0.36	1.16 ± 0.34^b^	1.06 ± 0.29c,d	<0.001
Triglycerides (mmol/l)	1.20 (0.90–1.60)	1.60 (1.10–2.20)	1.80 (1.20–2.40)c,d	<0.001
ApoA-I (g/l)	1.48 ± 0.25	1.45 ± 0.24	1.39 ± 0.22^c^	0.002
ApoB (g/l)	1.10 ± 0.24	1.16 ± 0.24	1.14 ± 0.26	0.14
Low-grade inflammation score (*Z*-score)	−0.19 ± 0.98	0.08 ± 0.93^a^	0.33 ± 1.01^c^	<0.001
Cholesterol efflux capacity	1.35 ± 0.30	1.34 ± 0.30	1.31 ± 0.29	0.42

Data are given as mean ± SD or median (interquartile range). Apo, apolipoprotein; BMI, body mass index; HbA1c, glycated hemoglobin; HDL, high density lipoproteins; LDL, low density lipoproteins; MetS, metabolic syndrome. Inflammation markers were available in 287 NGM, 126 IGM and 134 T2DM subjects. Low-grade inflammation score is expressed in Z-score of the whole study population. *Insulin was used in 12 T2DM subjects. *P*-value by ANOVA or chi-square test. ^a^*P* < 0.05 from NGM; ^b^*P* < 0.01 from NGM; ^c^*P* < 0.001 from NGM; ^d^*P* < 0.05 from IGM; ^e^*P* < 0.01 from IGM; ^f^*P* < 0.001 from IGM.

**Table 2:** Clinical characteristics, plasma glucose, glycated hemoglobin (HbA1c), plasma lipids, apolipoproteins (apos), inflammation marker score and cholesterol efflux in 552 subjects according to metabolic syndrome (MetS) classification.

	No MetS (n = 255)	MetS (n = 297)	*P*-value
Age (years)	59 ± 7	60 ± 7	0.033
Sex (men/women)	150/105	189/108	0.28
Glucose metabolism status (NGM/IGM/T2DM)	196/39/20	92/87/118	<0.001
Systolic blood pressure (mm Hg)	135 ± 20	145 ± 17	<0.001
Diastolic blood pressure (mm Hg)	79 ± 9	84 ± 9	<0.001
Waist circumference (cm)	93 ± 10	105 ± 11	<0.001
BMI (kg/m^2^)	26.5 ± 3.4	30.3 ± 4.3	<0.001
Current smoking (yes/no/unknown)	46/202/7	65/227/5	0.40
Alcohol consumption (gram/day)	10 (3–25)	7 (1–20)	0.46
Cardiovascular disease (yes/no)	51/204	106/191	<0.001
Glucose lowering drugs (yes/no)*	8/247	63/234	0.001
Lipid-modifying drugs (yes/no)	31/224	75/222	<0.001
Antihypertensive drugs (yes/no)	65/190	151/148	<0.001
Glucose (mmol/l)	5.4 ± 0.8	6.6 ± 1.7	<0.001
HbA1c (%)	5.7 ± 0.5	6.2 ± 0.9	<0.001
Total cholesterol (mmol/l)	5.14 ± 0.92	5.30 ± 1.04	0.066
Non-HDL cholesterol (mmol/l)	3.75 ± 0.94	4.27 ± 1.02	<0.001
LDL cholesterol (mmol/l)	3.25 ± 0.87	3.36 ± 0.86	0.15
HDL cholesterol (mmol/l)	1.39 ± 0.34	1.02 ± 0.25	<0.001
Triglycerides (mmol/l)	1.00 (0.80–1.40)	1.90 (1.40–2.20)	<0.001
ApoA-I (g/l)	1.55 ± 0.24	1.37 ± 0.21	<0.001
ApoB (g/l)	1.06 ± 0.23	1.19 ± 0.24	<0.001
Low-grade inflammation score (*Z*-score)	−0.30 ± 0.93	0.26 ± 0.99	<0.001
Cholesterol efflux capacity	1.38 ± 0.30	1.29 ± 0.28	<0.001

Data are given as mean ± SD or median (interquartile range). Apo, apolipoprotein; NGM, normal glucose metabolism; IGM, impaired glucose metabolism; T2DM, type 2 diabetes mellitus; BMI, body mass index; HbA1c, glycated hemoglobin; HDL, high density lipoproteins; LDL, low density lipoproteins. Inflammation markers were available in 293 subjects with MetS and in 254 subjects without MetS. Low-grade inflammation score is expressed in Z-score of the whole study population. *Insulin was used in 4 subjects without MetS and in 8 subjects with MetS.

**Table 3:** Relationships of cholesterol efflux capacity with clinical variables, metabolic syndrome (MetS) components, apolipoproteins (apos) and low-grade inflammation score in 552 subjects according to glucose metabolism status (normal glucose metabolism [NGM], impaired glucose metabolism [IGM] and Type 2 diabetes mellitus [T2DM]) and MetS classification.

	All subjects (n = 552)	NGM (n = 288)	IGM (n = 126)	T2DM (n = 138)	no MetS (n = 255)	MetS (n = 297)
Age	0.035	0.088	−0.063	0.101	0.038	0.069
Systolic blood pressure	0.024	0.082	−0.067	0.106	0.049	0.0.95
Alcohol consumption	0.129**	0.008	0.268**	0.178*	0.034	0.191**
Waist circumference	−0.034	−0.007	−0.033	−0.010	0.026	0.077
Glucose	−0.014	−0.075	0.039	0.100	0.046	0.062
HbA1c	−0.040	−0.030	−0.094	0.013	0.050	−0.010
Non-HDL cholesterol	0.026	0.016	0.003	0.053	0.041	0.095
HDL cholesterol	0.367***	0.372***	0.420***	0.301***	0.365***	0.317***
Triglycerides	−0.048	−0.056	−0.122	0.032	−0.023	0.096
ApoA-I	0.382***	0.365***	0.378***	0.401***	0.339***	0.368***
ApoB	−0.017	−0.034	−0.045	0.035	0.011	0.037
Low-grade inflammation score	−0.125**	−0.075	−0.139	−0.187*	0.009	−0.162**

Sex-adjusted partial correlation coefficients are shown. Apo, apolipopotein; HDL, high density lipoproteins; HbA1c, glycated hemoglobin. Inflammation markers were available in 547 subjects. Triglycerides are log transformed. *P < 0.05; **P ≤ 0.01; ***P ≤ 0.001.

**Table 4:** Multivariable linear regression analyses demonstrating relationships of cholesterol efflux with glucose metabolism status (normal glucose metabolism [NGM], impaired glucose metabolism [IGM] and Type 2 diabetes mellitus [T2DM]) and metabolic syndrome (MetS) in 552 subjects.

	Model 1		Model 2	
	β	*P*-value	β	*P*-value
Age	0.047	0.27	0.044	0.32
Sex (men vs. women)	−0.124	0.003	−0.168	<0.001
Glucose tolerance category				
IGM vs. NGM	0.037	0.43	0.026	0.57
T2DM vs. NGM	0.019	0.70	0.022	0.71
MetS (yes/no)	−0.169	<0.001	−0.150	0.002

β: standardized regression coefficient.

Model 1: adjusted for age and sex.

Model 2: additionally adjusted for current smoking, alcohol consumption, cardiovascular disease, glucose lowering drugs, lipid modifying drugs and antihypertensive medication.

**Table 5:** Multivariable linear regression analyses demonstrating relationships of cholesterol efflux with high density lipoprotein (HDL) cholesterol, apolipoprotein A-I (apoA-I) and low-grade inflammation score in 293 subjects with metabolic syndrome (MetS) (A) and in 254 subjects without MetS (B).

	Model 1		Model 2		Model 3		Model 4	
	β	*P*-value	β	*P*-value	β	*P*-value	β	*P*-value
(A) MetS (n = 293)								
Age	0.053	0.35	0.049	0.40	0.076	0.17	0.067	0.24
Sex (men vs. women)	−0.029	0.63	−0.084	0.20	−0.003	0.96	−0.048	0.46
HDL cholesterol	0.305	<0.001	0.295	<0.001				
ApoA-I					0.363	<0.001	0.356	<0.001
Low-grade inflammation score	−0.144	0.013	−0.156	0.007	−0.144	0.010	−0.151	0.009
(B) no MetS (n = 254)								
Age	−0.006	0.92	0.002	0.97	−0.009	0.88	−0.006	0.92
Sex (men vs. women)	0.040	0.55	0.042	0.56	0.023	0.73	0.033	0.66
HDL cholesterol	0.414	<0.001	0.411	<0.001				
ApoA-I					0.375	<0.001	0.376	<0.001
Low-grade inflammation score	0.079	0.197	0.105	0.096	0.045	0.46	0.069	0.28

β: standardized regression coefficient.

Models 1 include: age and sex, HDL cholesterol and low-grade inflammation score.

Models 2: additionally adjusted for current smoking, alcohol consumption, cardiovascular disease, glucose lowering drugs, lipid modifying drugs and antihypertensive medication.

Models 3 include: age and sex, apoA-I and low-grade inflammation score.

Models 4: additionally adjusted for current smoking, alcohol consumption, cardiovascular disease, glucose lowering drugs, lipid modifying drugs and antihypertensive medication.
