# Supplementary material for: Impaired HDL cholesterol efflux in metabolic syndrome is unrelated to glucose tolerance status: the CODAM study
Source: Sci Rep. 2016 Jun 8;6:27367. doi: 10.1038/srep27367 (PMC4897620; doi:10.1038/srep27367)
Supplement: Supplementary Information [file srep27367-s1.doc]

**Supplementary Information**

**Impaired HDL cholesterol efflux in metabolic syndrome is unrelated to glucose tolerance status: the CODAM study**

Wijtske Annema, Arne Dikkers, Jan Freark de Boer, Marleen M.J. van Greevenbroek, Carla J.H. van der Kallen, Casper G. Schalkwijk, Coen D.A. Stehouwer, Robin P.F. Dullaart, Uwe J.F. Tietge

**Supplementary Table I**. Comparison of cholesterol efflux capacity in patients with and without cardiovascular disease (CVD) stratified for presence or absence of metabolic syndrome (MetS).

|  | **I (n=203)** | **II (n=51)** | **III (n=190)** | **IV (n=103)** |  |
| --- | --- | --- | --- | --- | --- |
|  | **MetS- CVD-** | **MetS- CVD+** | **MetS+ CVD-** | **MetS+ CVD+** | **ANOVA** *P*-value |
| **CEC** | 1.39 ±0.30* | 1.37 ± 0.29 | 1.29 ± 0.28 | 1.28 ± 0.29 | 0.003 |

CEC: cholesterol efflux capacity. Data presented in mean ± SD. **P*<0.05 from III and IV; I vs. II: ns; III vs. IV: ns.

**Supplementary Table II**. Multivariable linear regression analyses demonstrating relationships of cholesterol efflux with high density lipoprotein (HDL) cholesterol, apolipoprotein A-I (apoA-I) and inflammation markers in all subjects (**A**), 287 subjects with normal glucose metabolism (NGM) (**B**), 126 subjects with impaired glucose metabolism (IGM) (**C**) and 134 Type 2 diabetes mellitus patients (T2DM) (**D**).

| **A All**  (n=547) | **Model 1** |  | **Model 2** |  | **Model 3** |  | **Model 4** |  |
| --- | --- | --- | --- | --- | --- | --- | --- | --- |
|  | **ß** | ***P*-value** | **ß** | ***P*-value** |  | ***P*-value** |  | ***P*-value** |
| **Age** | 0.028 | 0.68 | 0.023 | 0.59 | 0.033 | 0.81 | 0.027 | 0.52 |
| **Sex**  (men vs. women) | -0.005 | 0.13 | -0.030 | 0.52 | 0.006 | 0.89 | 0.008 | 0.87 |
| **Glucose tolerance category**  IGM vs NGM  T2DM vs. NGM | 0.044  0.057 | 0.30  0.19 | 0.038  0.064 | 0.38  0.25 | 0.013  0.021 | 0.76  0.63 | 0.008  0.022 | 0.85  0.69 |
| **HDL cholesterol** | 0.39 | <0.001 | 0.379 | <0.001 |  |  |  |  |
| **ApoA-I** |  |  |  |  | 0.391 | <0.001 | 0.391 | <0.001 |
| **Low-grade inflammation score** | -0.042 | 0.33 | -0.038 | 0.38 | -0.063 | 0.13 | -0.055 | 0.20 |

| **B NGM**  (n=287) | **Model 1** |  | **Model 2** |  | **Model 3** |  | **Model 4** |  |
| --- | --- | --- | --- | --- | --- | --- | --- | --- |
|  | **ß** | ***P*-value** | **ß** | ***P*-value** |  | ***P*-value** |  | ***P*-value** |
| **Age** | 0.040 | 0.48 | 0.027 | 0.64 | 0.034 | 0.56 | 0.024 | 0.69 |
| **Sex**  (men vs. women) | 0.003 | 0.97 | 0.021 | 0.74 | -0.005 | 0.93 | 0.027 | 0.68 |
| **HDL cholesterol** | 0.402 | <0.001 | 0.425 | <0.001 |  |  |  |  |
| **ApoA-I** |  |  |  |  | 0.384 | <0.001 | 0.413 | <0.001 |
| **Low-grade inflammation score** | 0.020 | 0.73 | 0.047 | 0.44 | -0.003 | 0.96 | 0.024 | 0.69 |

| **C IGM**  (n=126) | **Model 1** |  | **Model 2** |  | **Model 3** |  | **Model 4** |  |
| --- | --- | --- | --- | --- | --- | --- | --- | --- |
|  | **ß** | ***P*-value** | **ß** | ***P*-value** |  | ***P*-value** |  | ***P*-value** |
| **Age** | -0.105 | 0.20 | -0.116 | 0.16 | -0.063 | 0.45 | -0.081 | 0.34 |
| **Sex**  (men vs. women) | 0.130 | 0.17 | 0.047 | 0.64 | 0.122 | 0.22 | 0.048 | 0.72 |
| **HDL cholesterol** | 0.460 | <0.001 | 0.420 | <0.001 |  |  |  |  |
| **ApoA-I** |  |  |  |  | 0.415 | <0.001 | 0.371 | <0.001 |
| **Low-grade inflammation score** | -0.039 | 0.66 | -0.018 | 0.85 | -0.086 | 0.33 | -0.055 | 0.55 |

| **D T2DM**  (n=134) | **Model 1** |  | **Model 2** |  | **Model 3** |  | **Model 4** |  |
| --- | --- | --- | --- | --- | --- | --- | --- | --- |
|  | **ß** | ***P*-value** | **ß** | ***P*-value** |  | ***P*-value** |  | ***P*-value** |
| **Age** | 0.088 | 0.29 | 0.083 | 0.36 | 0.108 | 0.17 | 0.083 | 0.333 |
| **Sex**  (men vs. women) | -0.103 | 0.23 | -0.176 | 0.068 | 0.055 | 0.51 | -0.102 | 0.29 |
| **HDL cholesterol** | 0.286 | 0.001 | 0.260 | 0.006 |  |  |  |  |
| **ApoA-I** |  |  |  |  | 0.418 | <0.001 | 0.407 | <0.001 |
| **Low-grade inflammation score** | -0.143 | 0.087 | -0.179 | 0.039 | -0.145 | 0.066 | -0.161 | 0.052 |

ß: standardized regression coefficient.

Models 1 include: age and sex, HDL cholesterol and low-grade inflammation score.

Models 2: additionally adjusted for current smoking, alcohol consumption, cardiovascular disease, glucose lowering drugs, lipid modifying drugs and antihypertensive medication.

Models 3 include: age and sex, apoA-I and low-grade inflammation score.

Models 4: additionally adjusted for current smoking, alcohol consumption, cardiovascular disease, glucose lowering drugs, lipid modifying drugs and antihypertensive medication.

**Supplementary Table III**. Distribution of study participants over the different glucose tolerance and metabolic syndrome (MetS) categories.

|  | **NGM** | **IGM** | **T2DM** |  |
| --- | --- | --- | --- | --- |
| **MetS -** | 196 | 39 | 20 | 255 |
| **MetS +** | 92 | 87 | 118 | 297 |
|  | 288 | 126 | 138 | 552 |

Normal glucose metabolism (NGM), impaired glucose metabolism (IGM) and Type 2 diabetes mellitus (T2DM)
